# Nanoparticle-Based Visual Detection of Amplified DNA for Diagnosis of Hepatitis C Virus

**DOI:** 10.3390/bios12090744

**Published:** 2022-09-09

**Authors:** Soo-Kyung Kim, Yoon-Hee Oh, Dae-Hyun Ko, Heungsup Sung, Heung-Bum Oh, Sang-Hyun Hwang

**Affiliations:** 1 Department of Laboratory Medicine, Ewha Womans University College of Medicine, Seoul 07985, Korea; 2 Department of Laboratory Medicine, Asan Medical Center, University of Ulsan College of Medicine, Seoul 05505, Korea

**Keywords:** DNA detection, molecular POCTs, biosensing, paper-based speedy separation, nanoparticle

## Abstract

Rapid, simple, and inexpensive diagnostic point-of-care tests (POCTs) are essential for controlling infectious diseases in resource-limited settings. In this study, we developed a new detection system based on nanoparticle–DNA aggregation (STat aggregation of tagged DNA, STAT-DNA) to yield a visual change that can be easily detected by the naked eye. This simplified optical detection system was applied to detect hepatitis C virus (HCV). Reverse transcription-polymerase chain reaction (RT-PCR) was performed using primers labeled with biotin and digoxigenin. Streptavidin-coated magnetic particles (1 μm) and anti-digoxigenin antibody-coated polystyrene particles (250–350 nm) were added to form aggregates. The limit of detection (LoD) and analytical specificity were analyzed. The STAT-DNA results were compared with those of the standard real-time PCR assay using serum samples from 54 patients with hepatitis C. We achieved visualization of amplified DNA with the naked eye by adding nanoparticles to the PCR mixture without employing centrifugal force, probe addition, incubation, or dilution. The LoD of STAT-DNA was at least 10^1^ IU/mL. STAT-DNA did not show cross-reactivity with eight viral pathogens. The detection using STAT-DNA was consistent with that using standard real-time PCR.

## 1. Introduction

Hepatitis C, which is caused by hepatitis C virus (HCV), is a global health concern. It is associated with high morbidity and mortality, especially in low-income settings [1]. Globally, it is estimated that 71 million individuals have chronic HCV infection; the annual mortality is 399,000 individuals [2].

HCV RNA detection using real-time polymerase chain reaction (PCR) is the gold standard for the diagnosis of HCV infection and is also important in the management of therapeutic regimens [3]. With the development of cost-effective and efficacious oral antivirals, HCV infection can be cured in more than 95% of cases with timely diagnosis and treatment [4]. However, it is estimated that up to 80% of HCV-infected people are not aware of their condition, particularly in low-to-middle-income areas [5]. Thus, increased diagnosis using an affordable diagnostic point-of-care test (POCT) and appropriate treatment are essential to achieve the World Health Organization (WHO) 2030 elimination goals [1].

Owing to the lack of infrastructure and/or trained operators, centralized laboratory testing is not feasible for patients with hepatitis C in resource-limited settings [6]; in such cases, an affordable and rapid POCT would be particularly useful [7]. However, in resource-limited settings, molecular POCTs should have certain qualities for easy usage, for example, visual detection by the naked eye, short (less than 30 min) incubation time, no or minimal washing process for signal generation, affordability, and high reliability with no additional equipment requirements [8]. We previously developed a visual detection system involving the paper-based speedy separation of amplified DNA (PASS-DNA) [9]; however, it requires both paper and particles (beads) because it separates DNA-bound particles from those not bound to DNA.

Nanoparticles are extensively used in optical sensing approaches such as fluorescence, electrochemiluminescence (ECL), surface plasmon resonance (SPR), and surface-enhanced Raman scattering (SERS) for viral detection [10].

Colorimetric biosensors using gold nanoparticles (AuNPs) are easy to use, and the detection is through the visualization of a color change resulting from plasmonic interactions between the particles [11]. AuNPs are the preferred metal in most SPR sensors owing to their unique optical properties and biocompatibility [12]. Therefore, these reactions are promising for diagnosing various infectious diseases [13,14].

In the plasmonic colorimetric assay, the shifts in the plasmon bands due to the aggregation of nanoparticles in a size-dependent manner can cause visible color transitions from red to blue and blue to red [15]. It is strongly dependent on covalent or non-covalent interactions and external conditions such as temperature, pH, and buffer solution [15].

The functionalization of the surface significantly affects the performance of AuNP-based colorimetry. The immobilization of the ligand on the surface leads to a negative effect on molecular interactions [12,16].

The plasmonic colorimetric method has a poor resolution for naked-eye detection, especially for low target concentrations; in addition, color readouts through visual inspections rely on mono-color changes and may lead to false-positive/-negative results [15].

In this study, we aimed to develop a simple method for visualizing amplified DNA without employing any other separation method, such as paper [9] or a centrifugal device/column [17]. We were able to detect amplified DNA using only the naked eye by adding nanoparticles without plasmonic interactions between them. Additional centrifugation, probe conjugation, long incubation, signal generation, or dilution was not required. This simplified optical detection system for target DNA was evaluated using HCV RNA to validate this methodology.

## 2. Materials and Methods

### 2.1. Nanoparticle-Based Visual Detection of Amplified DNA (STat Aggregation of Tagged DNA, STAT-DNA)

**Figure 1:**
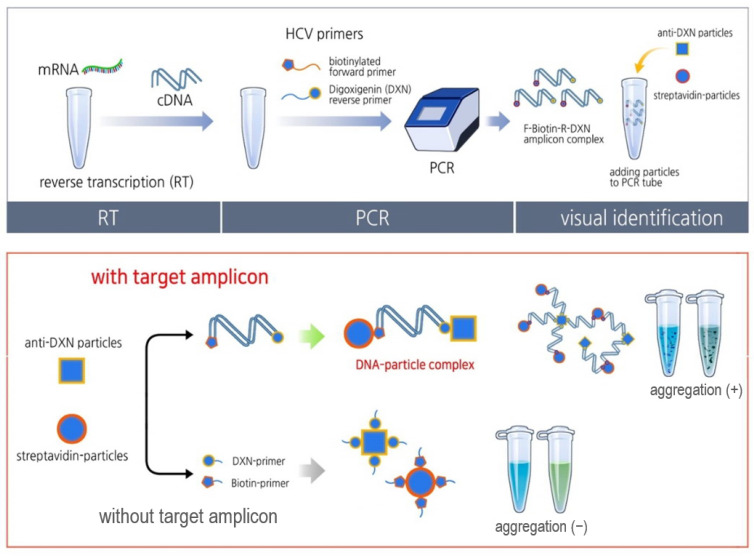
Schematic representation of the nanoparticle-based visual detection of amplified DNA (STat Aggregation of Tagged DNA, STAT-DNA) for the detection of hepatitis C virus (HCV) RNA. The target HCV amplicons were amplified using biotin- or digoxigenin (DXN)-labeled primers and captured using streptavidin particles (brown magnetic particles or blue polystyrene particles) and anti-DXN nanoparticles. The amplicon–particle complex produces aggregations within a minute; these can be visualized using the naked eye.

The principle of the STAT-DNA methodology is illustrated in Figure 1.

The final optimized protocol included the following steps: Nucleic acid amplification was carried out with forward and reverse primers labeled with biotin and digoxigenin, respectively, using reverse transcription-polymerase chain reaction (RT-PCR). A mixture of 2 μL of streptavidin-coated magnetic bead suspension (1 μm, 10 mg/mL Dynabeads MyOne Streptavidin C1; Life Technologies, Grand Island, NY, USA) and 1 μL of anti-digoxigenin antibody-coated polystyrene particles (250–350 nm, 5 mg/mL anti-digoxigenin alpha-donor beads in PBS pH 7.2; PerkinElmer, Waltham, MA, USA) was added to PCR tubes containing amplified DNA. Following the visual detection of particle aggregation, a low ionic strength solution (LISS) buffer (BLISS; 60 µL; Ortho-Clinical Diagnostics, Raritan, NJ, USA) was added to differentiate between positive and negative results, which was otherwise difficult to achieve due to the small PCR volume (25 µL).

To evaluate the effect of particle size on STAT-DNA, the same experiment was performed using streptavidin-coated blue polystyrene particles (300–390 nm, SPHERO; Spherotech, Lake Forest, IL, USA) instead of streptavidin-coated brown magnetic particles (Dynabeads).

### 2.2. Sample Collection

Serum samples were collected from HCV-infected patients at the Asan Medical Center (Seoul, Korea) from May 2018 to October 2020. After the routine HCV RNA test using the Roche Cobas 6800 system (Cobas HCV; Roche Molecular Diagnostics, Rotkreuz, Switzerland), each leftover or residual serum sample was aliquoted and stored at −80 °C. This study was approved by the Asan Medical Center Institutional Review Board (IRB No. 2017-1162).

### 2.3. RNA Extraction and cDNA Synthesis

HCV RNA was extracted from clinical samples using the QIAamp MinElute Virus Spin Kit (Qiagen, Germantown, MD, USA) according to the manufacturer’s protocol and stored at −80 °C until further analysis. HCV cDNA synthesis was performed using the Roche Transcriptor First Strand cDNA Synthesis Kit (Roche, Mannheim, Germany) according to the manufacturer’s instructions. Briefly, 5 μL of isolated RNA was mixed with 20 μL of the RT reaction mix containing 4 μL of 5× reaction mix, 0.5 μL of Transcriptor Reverse Transcriptase (Roche), 2 μL of random hexamer (20 pmol), 2 μL of deoxynucleotide mix, 0.5 μL of Protector RNase Inhibitor, and 6 μL of nuclease-free water (Roche). cDNA synthesis was carried out in an FX thermocycler (Bio-Rad, Hercules, CA, USA) (incubation conditions: 1 cycle at 25 °C for 10 min, 55 °C for 30 min, and 85 °C for 5 min). cDNA samples were stored at −80 °C until further PCR analysis.

### 2.4. PCR Amplification

The RT-PCR primers for simple visual detection of HCV mRNA were modified from a previous study using a pair of specially designed primers for the 5′-untranslated region (5′-UTR) of HCV [18]. The generated product of the HCV amplicon was 157 bp long (Table 1). 

The forward primer was biotinylated at the 5′ end, and the reverse primer was labeled with digoxigenin at the 5′ end. They were suitable for capture by streptavidin-coated magnetic particles and anti-digoxigenin antibody-coated nanoparticles, respectively.

The RT-PCR was performed according to the manufacturer’s instructions using the QIAGEN Taq PCR Master Mix Kit (Qiagen). Briefly, 5 μL of cDNA was amplified in a 25 μL reaction mixture containing 12.5 μL of QIAGEN Taq PCR Master Mix (Qiagen), 1 μL of biotinylated forward primer, 1 μL of digoxigenin-labeled reverse primer, and distilled water. Thermal cycling was performed on an Applied Biosystems 7500 instrument (Applied Biosystems, Foster City, CA, USA) under the following conditions: 94 °C for 5 min; 40 cycles at 95 °C for 30 s, 56 °C for 30 s, and 72 °C for 30 s; and final extension at 72 °C for 10 min. Agarose gel (2%) electrophoresis was used to detect the HCV PCR amplicons.

The performance of the detection system was evaluated in comparison with a clinical real-time PCR test (Cobas HCV) for the detection and quantification of HCV RNA.

## 3. Results

### 3.1. Assay Optimization: Difference in Aggregations of Amplicon-Captured Versus Non-Amplicon-Captured Particles in STAT-DNA

To identify aggregations produced by the particle–DNA complexes at an early stage, a 5′-biotin- and 3′-digoxigenin-labeled HCV-specific sequence with a spacer molecule (5′-biotin-CCG GGG CAC TCG CAA GCA CCC-iSp18-digoxigenin-3′) mimicking the PCR amplicon was prepared for the particle−HCV sequence complex. The PCR amplicons could cause contamination in the laboratory; therefore, we should not observe aggregations of PCR amplicons–particles under the microscope. Spacer molecules provide greater lateral separation of DNA on the particles, reducing hybridization issues due to steric hindrance [19].

**Figure 2:**
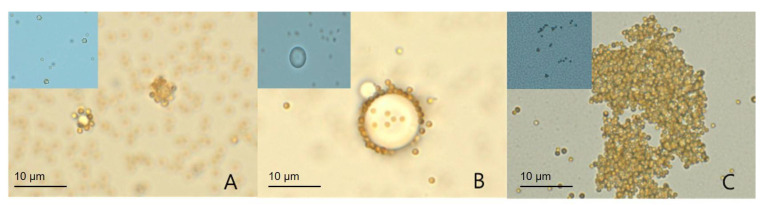
Pilot test for the identification of particle–DNA sequence complexes. Streptavidin-coated magnetic particles were tested with three different kinds of anti-digoxigenin particles: (**A**) 2.8 µm SPHERO anti-digoxigenin magnetic particles (Spherotech); (**B**) 10.4 µm SPHERO anti-digoxigenin polystyrene particles (Spherotech); and (**C**) 250–350 nm alpha latex particles (PerkinElmer). The streptavidin-coated magnetic particle–DNA–alpha particle complex (**C**) showed the most prominent aggregation. No aggregations were observed without DNA sequence addition (upper left panels in blue background). All images were obtained at ×1000 magnification (optical microscope).

In this pilot stage, streptavidin-coated magnetic particles were tested with several anti-digoxigenin particles (2.8 µm SPHERO magnetic particles, Spherotech; 10.4 µm SPHERO polystyrene particles, Spherotech; 250–350 nm alpha latex particles, PerkinElmer) to identify particle–DNA sequence complexes. The combination of streptavidin-coated magnetic particles and anti-digoxigenin alpha latex particles showed prominent, large aggregations with DNA sequences (Figure 2).

**Figure 3:**
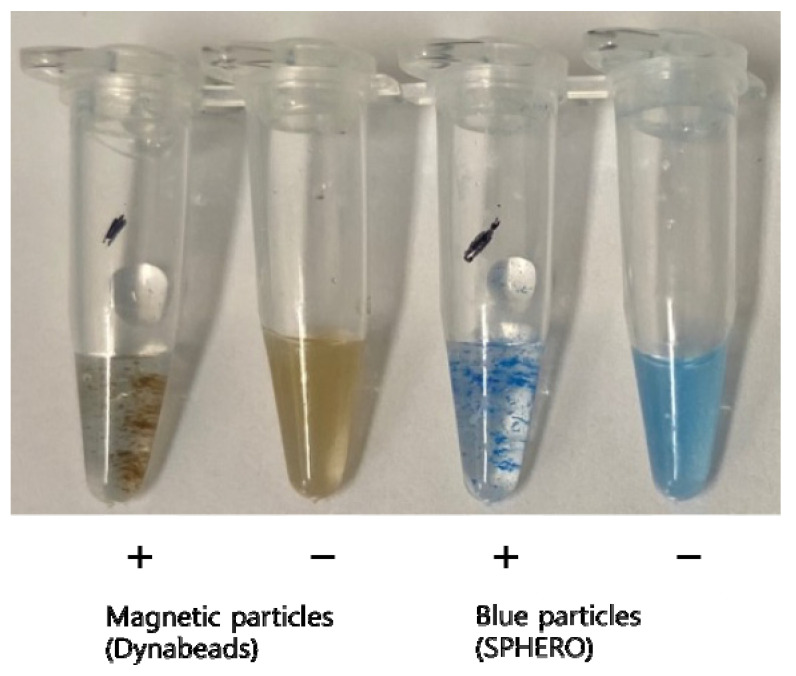
Evaluation of different sized streptavidin-coated particles (blue polystyrene particles of ~300 nm and magnetic particles of ~1 µm) for visual detection of hepatitis C virus (HCV) amplicons. Regardless of particle size, aggregations were visually detected within a minute after adding the nanoparticles to the tubes containing HCV cDNA amplicons (+). No aggregation was observed in the absence of the target amplicons (−).

After adding nanoparticles to the PCR tubes, particles started to aggregate in the presence of the target amplicons within 1 min, whereas no such aggregation was observed in the absence of the target amplicons (Figure 3).

Among commercially available anti-digoxigenin antibody-coated particles, we selected blue polystyrene particles (250–350 nm anti-digoxigenin alpha-donor beads, PerkinElmer; 5 mg/mL) to capture the digoxigenin-labeled reverse primer.

The concentration of the nanoparticles was optimized for the clear visualization of amplicon aggregation. The optimal concentration of the nanoparticles was determined to be 2 μL of streptavidin-coated magnetic or blue polystyrene particles (10 mg/mL) and 1 μL of anti-digoxigenin antibody-coated polystyrene particles (5 mg/mL) (Figure S1). 

### 3.2. Reproducibility and Detection Limit

**Figure 4:**
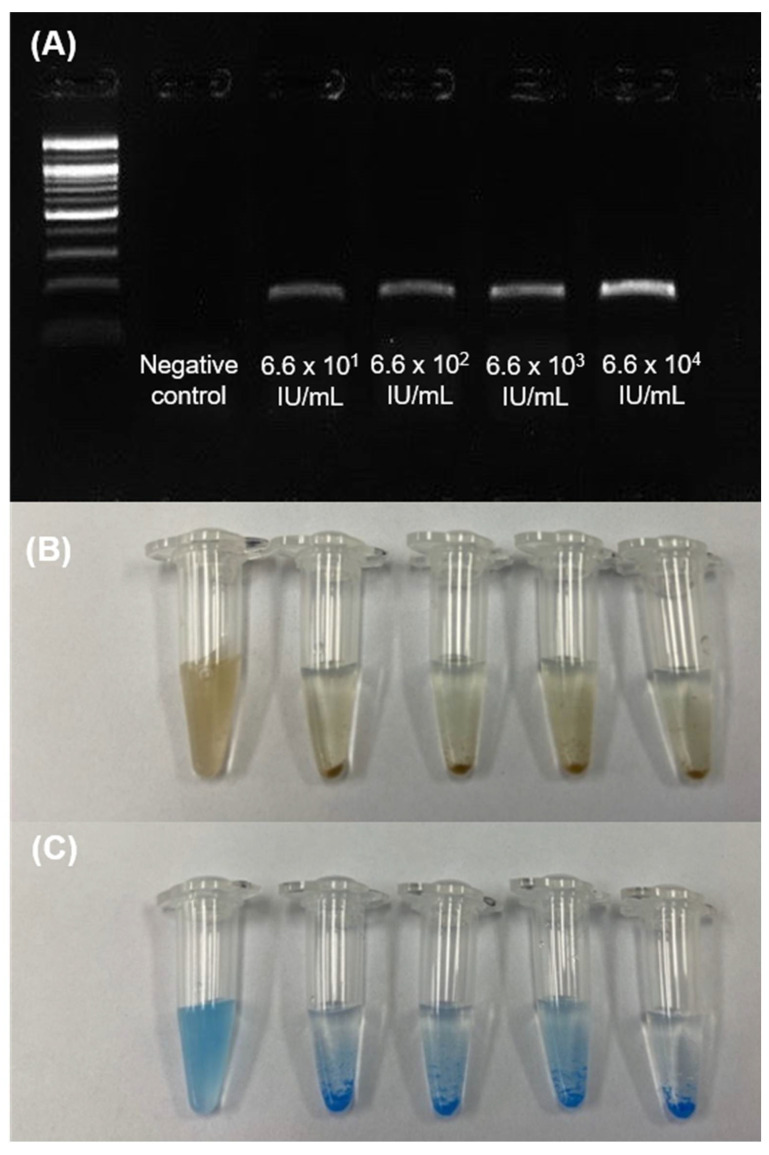
Determination of the precision and limit of detection of STAT-DNA for the detection of serially diluted hepatitis C virus (HCV) cDNA at ~10^1^, ~10^2^, ~10^3^, and ~10^4^ IU/mL. (**A**) Gel electrophoresis of real-time PCR HCV sequences. Corresponding STAT-DNA results using streptavidin-coated magnetic particles (**B**) and streptavidin-coated blue particles (**C**). Large aggregations were noted in the tubes containing HCV amplicons, whereas no aggregations were obtained in the negative control tubes.

Precision and limit of detection (LoD) were analyzed using serially diluted HCV cDNA samples at ~10^1^, ~10^2^, ~10^3^, and ~10^4^ IU/mL. Reproducible results were obtained by performing two runs of STAT-DNA for 5 days with the serially diluted samples. It showed 100% consistent results. The LoD of the STAT-DNA biosensor was determined by comparing the results with those of the conventional agarose gel detection of serially diluted PCR amplicons. The STAT-DNA biosensor was clearly observed at ~10^1^ IU/mL (Figure 4). Therefore, detection using STAT-DNA is comparable to that using conventional PCR and real-time PCR [20].

### 3.3. Comparison with Clinical Samples

**Figure 5:**
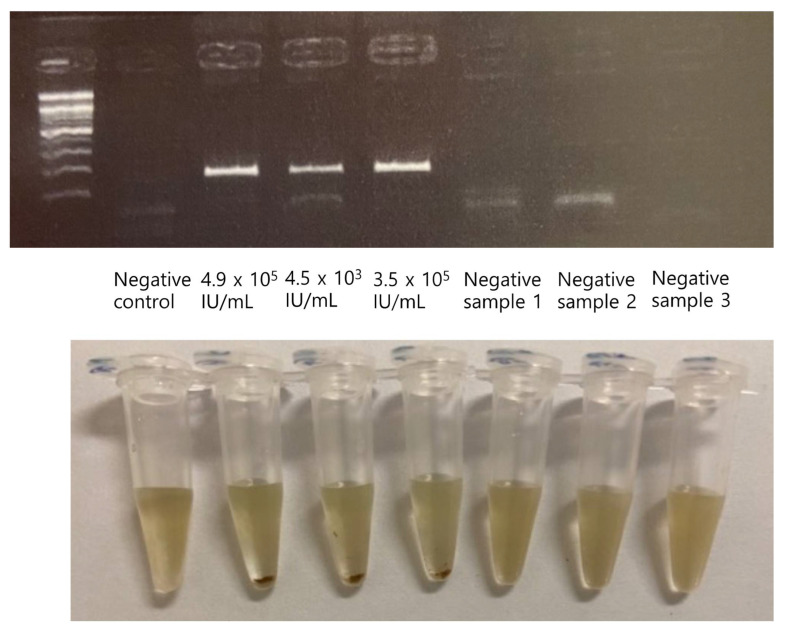
Representative results of the STAT-DNA using clinical samples. The results of STAT-DNA were compared with those of the Cobas HCV test using clinical samples. Three positive samples and three negative samples are shown ((**top**) gel electrophoresis of HCV PCR amplicons; (**bottom**) corresponding STAT-DNA results).

The results of STAT-DNA were compared to those of the Cobas HCV test. Among the 54 samples, 32 were identified as positive, with concentrations ranging from 1.4 × 10^2^ IU/mL to 1.2 × 10^7^ IU/mL; 22 were identified as negative for HCV using STAT-DNA. These results showed 100% concordance with the results of the Cobas HCV test. Analysis using the STAT-DNA method showed no discordant or unresolved samples (Figure 5).

### 3.4. Analytical Specificity

**Figure 6:**
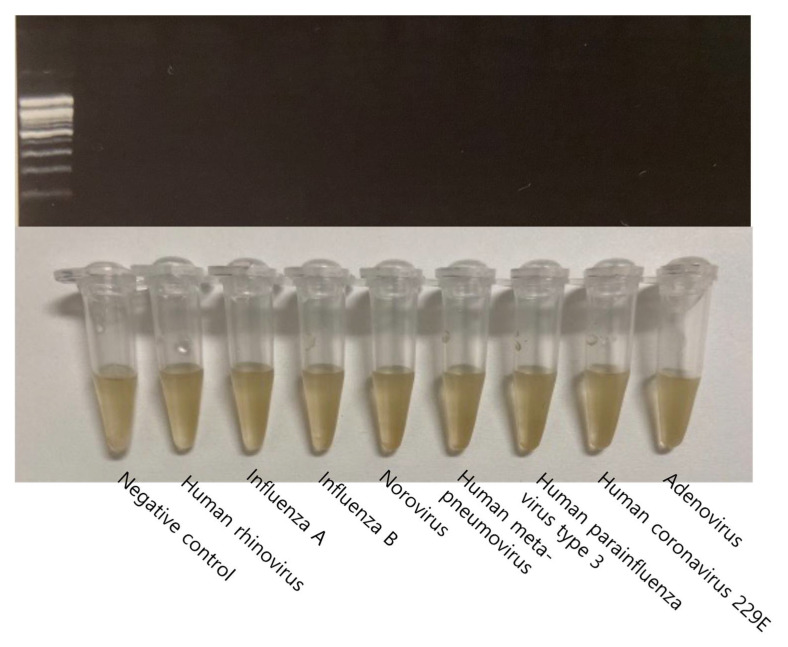
The analytical specificity of STAT-DNA. No cross-reactivity was observed for other viruses, i.e., human rhinovirus, influenza A virus, influenza B virus, norovirus, human metapneumovirus, human parainfluenza virus type 3, human coronavirus 229E, and adenovirus ((**top**) gel electrophoresis of HCV PCR amplicons; (**bottom**) corresponding STAT-DNA results).

Several viral strains were selected to evaluate the analytical specificity of the STAT-DNA system. No cross-reactivity was detected for the following eight viruses: human rhinovirus, influenza A virus, influenza B virus, norovirus, human metapneumovirus, human parainfluenza virus type 3, human coronavirus 229E, and adenovirus (Figure 6).

## 4. Discussion

STAT-DNA is a simple, rapid qualitative test with 100% sensitivity and specificity, with a detection limit of ~10 IU/mL. The AuNP-based biosensor developed by Shawky et al. detected 26 out of 28 samples, with a sensitivity and specificity of 93.3% and 100%, respectively [21]. The performance of our method was comparable to that of the AuNP-based biosensor developed by Shawky et al. [21]. The detection limit of our method was lower than that of the AuNP-based biosensor developed by Mohammed et al., showing a detection limit of 100 IU/mL [13]. To demonstrate the comparative performance of STAT-DNA, a comprehensive range of HCV loads was chosen to represent low to high viral loads (ranging from 1.4 × 10^2^ IU/mL to 1.2 × 10^7^ IU/mL). STAT-DNA showed good analytical specificity. There was no cross-reactivity with clinically important viruses.

The STAT-DNA system is one of the simplest visual detection systems among DNA sensors, especially when compared with lateral flow-based detection methods. Several studies combining DNA amplification and a lateral flow immunoassay (LFIA) to detect viral nucleic acids have been published [22]. However, the detection of amplified DNA using LFIA requires a complex sensor design for capturing oligos [23,24] or antibodies [25], along with sophisticated detection probes or sequence designs [26]. For hybridization with a target, a heating process is also required [26]. To detect captured DNA, signal detection steps, including fluorescent scanning [27] or visual RT-LAMP-CRISPR detection under a blue light illuminator, are required [28]. However, STAT-DNA does not require a lateral flow dipstick, membrane paper, a dispensing system for line dispensing, protein blockers, stabilizers, immobilization of the capture reagent in the membrane [29,30], or a detection procedure, such as a hybridization step with heating [26]. In our previous studies, several factors affected the migration of particles in the SPIN-DNA [17] and PASS-DNA [9] systems, such as Sepharose and intercalating dye concentration, centrifugal force, and Cy3 labeling. These steps are not required in the STAT-DNA system; therefore, visual detection was almost unbiased.

The STAT-DNA visualization technique does not require sophisticated instrumentation, immobilization techniques, or transduction technology, such as surface plasmon resonance (SPR) [31], electrochemical transduction [24], and surface-enhanced Raman scattering (SERS)-based biosensors [32].

There are several optical methods coupled with nucleic acid amplification techniques for the visual detection of analytes, such as fluorescence readouts [33], pH indicators [34], and gold nanoparticle (AuNP) aggregation [21]. AuNPs, which have properties such as SPR, undergo visible color changes [35,36]. Therefore, AuNP aggregation based on cross-linking, non-cross-linking, or unmodified charge-based aggregation induced by pH, ionic strength, catalytic DNA circuits, or charge-dependent processes has been widely employed in colorimetric assays for nucleic acid detection [21,35,37]. However, sophisticated methods are sometimes required to improve the sensitivity of AuNP-based colorimetry and to change the color of the colloidal solution [35,38].

One of the limitations of the plasmonic colorimetric method is the poor resolution of naked-eye detection, especially for low target concentrations, because color readouts through visual inspections rely on mono-color changes and may lead to false-positive/-negative results [15]. The STAT-DNA system clearly differentiated positive from negative samples at very low HCV concentrations. In addition, there is no need for a development process to enhance the color or signal because the nanoparticles are noticeable, and the spontaneous aggregation in the PCR tubes exempts the need for driving force or microfluidic operations.

Although PCR is labor-intensive and time-consuming, it is still a commonly used and recommended method for the detection of severe acute respiratory syndrome-coronavirus-2 (SARS-CoV-2) owing to its high sensitivity and specificity [39]. However, primer dimers can produce undesirable false positives unless an additional specific detection design is included (e.g., probe-based detection), especially in real-time PCR. STAT-DNA is minimally affected by the presence of primer dimers because nanoparticles cannot interact with sequences as short as primer dimers. 

Magnetic nanoparticle-based capture is widely used in DNA sensors owing to their good dispersion, low cost, easy separation, purification in buffer systems, and signal detection [40]. However, signal detection requires additional development processes for fluorescent, electrochemical, or chemiluminescent detection [22,41,42]. In the STAT-DNA system, we did not use magnetic nanoparticles for their magnetically controllable properties, such as aggregation, purification, and separation. The role of the brown magnetic particles in STAT-DNA is to facilitate naked-eye detection following aggregation. This is similar to the role of streptavidin-coated blue particles in the STAT-DNA system.

The amount of nanoparticles used in this study was relatively low (5–10 μg) as compared with that in the lateral flow method for visual detection. Chua et al. showed that the minimum amount of capture reagent required to generate an intense red line in the lateral flow method was 20 μg for both streptavidin-coated nanoparticles and anti-digoxigenin antibody-coated nanoparticles [43]. 

Our STAT-DNA test can be performed with a broad range of nano- (250–300 nm-sized anti-digoxigenin antibody-coated polystyrene particles) to micro-sized particles (1 μm-sized streptavidin-coated magnetic particles). To avoid several optimization steps, such as conjugation to nanoparticles, and to eliminate unexpected variability during optimization, we used commercially available antibody-coated or conjugated nanoparticles to capture biotin- or digoxigenin-labeled primers. Among the commercially available anti-digoxigenin antibody-coated particles, the alpha-donor particles successfully produced the aggregation of DNA amplicons with streptavidin-coated microparticles. Anti-digoxigenin alpha-donor particles were 250–350 nm in size [44], originally used in homogeneous (no wash) bead-based sandwich immunoassays. However, to the best of our knowledge, the STAT-DNA system is the first to use alpha-donor particles in DNA biosensors.

As a proof of concept, we used conventional two-step PCR for the amplification of nucleic acids. However, to overcome the use of the time-consuming two-step real-time PCR, isothermal DNA amplification technology can be used to provide a molecular diagnosis of infectious diseases at the point of care. Isothermal amplification methods (such as LAMP, recombinase polymerase amplification, and helicase-dependent amplification) [3,45] can also be used with our STAT-DNA visual detection system.

## 5. Conclusions

STAT-DNA is a nanoparticle-based and simple visual detection system for amplified DNA without any substantial requirements, such as a centrifugal device, gel column or membrane for separation, or fluorescent detection [46]. The detection sensitivity of the STAT-DNA system was comparable to that of the conventional clinical-grade RT-PCR analysis. With further improvements, such as the microfluidic combination of sample preparation, nucleic acid isolation, and amplification, STAT-DNA can be applied effectively in resource-limited settings [44]. 

## Figures and Tables

**Table 1:** Reverse transcription-polymerase chain reaction (PCR) primers for simple visual detection of hepatitis C virus RNA.

Primer	Sequence	Location	PCR Target Size
Forward	5′-biotin-TGCACGGTCTACGAGAC-3′	322–339	157 bp
Reverse	5′- digoxigenin-CGACCGGGTCCTTTCTTGGAT-3′	182–203	

## Data Availability

Data are available on request.

## Supplementary Materials

The following supporting information can be downloaded at: https://www.mdpi.com/article/10.3390/bios12090744/s1, Figure S1: Streptavidin-coated magnetic particles and anti-digoxigenin antibody-coated alpha particles were added at 0.5, 1.0, and 2.0 µL to produce a clear visualization of particle-amplicon aggregations.
