# Supplementary material for: Nanoparticle-Based Visual Detection of Amplified DNA for Diagnosis of Hepatitis C Virus
Source: Biosensors (Basel). 2022 Sep 9;12(9):744. doi: 10.3390/bios12090744 (PMC9496050; doi:10.3390/bios12090744)
Supplement: Supplementary file 1 [file biosensors-12-00744-s001.zip › biosensors-1870272-supplementary.pdf]

biosensors

Nanoparticle-Based Visual Detection of Amplified DNA for Diagnosis of Hepatitis C Virus

Soo-Kyung Kim [1], Yoon-Hee Oh [2], Dae-Hyun Ko [2], Heungsup Sung [2], Heung-Bum Oh [2] and Sang-Hyun Hwang [2,*]

[1] Department of Laboratory Medicine, Ewha Womans University College of Medicine, Seoul 07985, Korea
[2] Department of Laboratory Medicine, Asan Medical Center, University of Ulsan College of Medicine, Seoul 05505, Korea
* Correspondence: mindcatch@amc.seoul.kr; Tel.: +82-2-3010-4502

Streptavidin-coated magnetic particles	Anti-digoxigenin alpha particles (0.5 μL, 1.0 μL, or 2.0 μL were added to each tube, from left to right)	
	1 min after addition	2 min after addition
0.5 μL	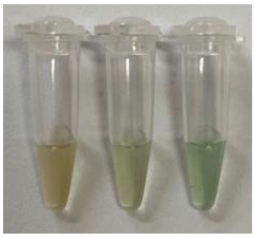	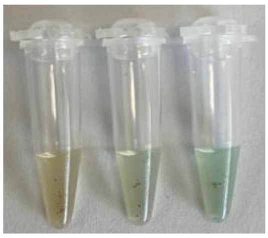
1.0 μL	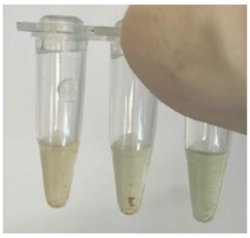	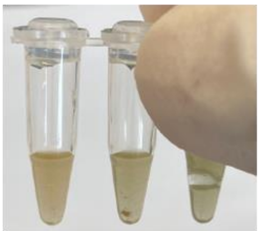
2.0 μL	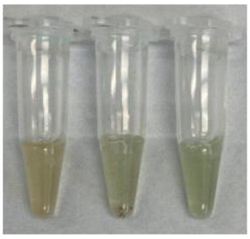	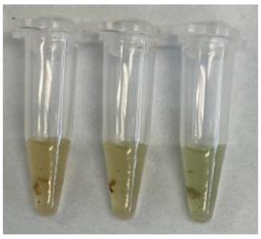

Figure S1. Streptavidin-coated magnetic particles and anti-digoxigenin antibody-coated alpha particles were added at 0.5, 1.0, and 2.0 μL to produce a clear visualization of particle-amplicon aggregations. All experiments were performed in triplicate, at least. Mixing 1 μL or 2 μL of streptavidin-coated magnetic particles with 1 μL of anti-digoxigenin polystyrene particles led to the formation of particles-HCV amplicon complexes within 1 min. Prominent aggregation of particles-amplicon complexes was observed after 2 min when using 2 μL of streptavidin-coated particles, regardless the concentration of anti-digoxigenin particles.
